# Malaysian burden of disease: years of life lost due to premature deaths

**DOI:** 10.1186/s12889-023-16309-z

**Published:** 2023-07-18

**Authors:** Wan-Fei Khaw, Yee Mang Chan, Nur Hamizah Nasaruddin, Nazirah Alias, LeeAnn Tan, Shubash Shander Ganapathy

**Affiliations:** Institute for Public Health, Ministry of Health Malaysia, Shah Alam, Selangor Malaysia

**Keywords:** Mortality, Premature, Burden of disease, Population health, Malaysia

## Abstract

**Background:**

In Malaysia, the previous mortality burden has been a significant concern, particularly due to the high prevalence of noncommunicable diseases (NCDs) as the leading cause of death. Estimates of mortality are key indicators for monitoring population health and determining priorities in health policies and health planning. The aim of this study was to estimate the disease burden attributed to 113 major diseases and injuries in Malaysia in 2018 using years of life lost (YLL) method.

**Methods:**

This study included all deaths that occurred in Malaysia in 2018. The YLL was derived by adding the number of deaths from 113 specific diseases and multiplying it by the remaining life expectancy for that age and sex group. Data on life expectancy and mortality were collected from the Department of Statistics Malaysia.

**Results:**

In 2018, there were 3.5 million YLL in Malaysia. Group II (NCDs) caused 72.2% of total YLL. Ischaemic heart disease was the leading cause of premature mortality among Malaysians (17.7%), followed by lower respiratory infections (9.7%), road traffic injuries (8.7%), cerebrovascular disease (stroke) (8.0%), and diabetes mellitus (3.9%).

**Conclusions:**

NCDs are a significant health concern in Malaysia and are the primary contributor to the overall burden of disease. These results are important in guiding the national health systems on how to design and implement effective interventions for NCDs, as well as how to prioritise and allocate healthcare resources. Key strategies to consider include implementing health promotion campaigns, adopting integrated care models, and implementing policy and regulatory measures. These approaches aim to enhance health outcomes and the managements of NCDs in Malaysia.

## Background

Burden of disease analysis aims to provide internationally comparable health status of populations based on a standardised concept [1]. Disability-adjusted life year (DALY) is an essential component of population health analysis, which acts as a composite metric that incorporates mortality and morbidity to assess the population’s overall health [2]. The DALY measures two components: years lived with disability (YLD, morbidity component) and years of life lost (YLL, mortality component). This measure calculates the years of healthy life lost to disease and death to quantify the actual impact of health conditions on a population. This information enables direct comparisons of various health conditions and helps in identifying which preventative and treatment strategies should be prioritised. As a result, disease burden measurements are utilised in both national and global health information systems to determine population health.

The Global Burden of Disease Study (GBD) provides a global overview of the disease burden [3], but studies of burden estimates in Malaysia have limitations in their data sources [4–6]. These challenges may include issues with disease registries, national health surveys, or other data sources, resulting in insufficient coverage, incomplete data, or inconsistencies in data collection methods. Also, many countries have conducted their own burden of disease studies [7–9]. The Malaysian Burden of Disease (MBOD) project, conducted by the Institute for Public Health, Ministry of Health Malaysia, aims to provide policymakers with accurate and statistically representative information on related areas using reproducible data and methods.

The Institute for Public Health Malaysia adopted and utilised GBD concept in MBOD. The first MBOD estimates were published in 2004 [10]. The MBOD study contributes to the input for public health priorities and policies. Over 20 years, the MBOD calculation methodology has evolved [11–13]. For example, the improvement of data availability and list of diseases enables the addition of more diseases in the recent MBOD. In 2001, the YLL in Malaysia estimated 1.7 million, which increased to 3.1 million by 2014. The top three leading causes of mortality were ischaemic heart disease, road traffic accidents, and cerebrovascular disease. These findings highlight the significant impact of these diseases on premature mortality.

YLL, a measure of the impact of a disease on a population, which is calculated by adding up the numbers of years that each individual who died from the disease would have lived if they had not died prematurely. Because data on YLD may not be available, some studies only focus on calculating the YLL component by using national vital statistics [4–6, 14–17]. In this particular study, the researchers aimed to provide an overview of the burden of disease in Malaysia, focusing on calculating the mortality component of the burden. Instead of determining the primary cause of death based on the number of deaths, this study calculates the YLL to estimate the impact of death on population health. The YLL metric prioritised high-impact diseases for which preventive, curative approaches should be emphasised to minimise the loss of life and increase life expectancy. Therefore, the present article comprehensively assessed the disease burden attributed to 113 major diseases and injuries in 2018 using the YLL method.

## Methods

### Selection of disease and injury category

The GBD study categorised diseases into three groups: group I (communicable disease, maternal, perinatal, nutritional conditions), group II (noncommunicable diseases) and group III (injuries). These three groups are further divided into 22 cause categories and classified into specific diseases at level 3. In Malaysia, a list of 113 specific diseases was selected and modified by public health experts to be more relevant to the country’s epidemiology situation [12].

### Estimation of premature mortality

Calculation of premature mortality in Malaysia in 2018 used methodologies developed by Murray and Lopez for the GBD [18] to calculate the YLL and the methodology of the MBOD was detailed [12, 13]. The YLL was calculated by multiplying the number of deaths for a specific disease category by the remaining life expectancy for a specific age and sex group. The formula is as follows:

YLL(c,s,a,t) = N(c,s,a,t) x LE(s,a,t)

Where:

N (c,s,a,t) is the number of deaths attributable to cause (c), for the specified age group (a), and sex (s), in year (t).

LE (s,a,t) is the remaining life expectancy for the specified age group (a), and sex (s), in year (t).

Mortality data from January to December 2018 were included in this study. Data on deaths, including sex, age and cause of death and ICD-10 codes were obtained from the Department of Statistics Malaysia (DOSM). National Registration Department Malaysia gathered mortality data through the vital registration system and DOSM assigned ICD-10 codes for each death. There are two categories of mortality data: medically certified deaths and non-medically certified deaths. Medically certified deaths occur in medical facilities and are certified by the attending physician as the cause of death. These deaths were assigned codes according to the 10th edition of ICD-10. In contrast, non-medically certified deaths happen outside medical facilities and are reported by deceased’s family to the local police, who also provide a non-expert opinion of the cause of death. These deaths usually were assigned death codes by the DOSM.

The mortality data with missing age and sex were minimal, less than 0.1% of the total. When missing values occurred, it was assigned to the age group or sex with the highest prevalence for the cause of death. Gross errors in the mortality data were determined and corrected through age- and sex-specific diseases check.

When there is not enough information to determine the true cause of death, death is classified with less informative codes, known as “garbage codes”. To address this, experts in the Burden of Disease analysed the garbage codes listed by the World Health Organization and GBD Study [12, 13]. Using cause specific mortality fractions derived via verbal autopsy, the ill-defined causes were then distributed among specific disease groups, specific cause categories or all cause categories.

The data on life expectancy was taken from life tables created by the DOSM using three-year death records and the mid-year population of 2018. The average life expectancy for each age and sex group was calculated based on the average age of death in that particular age range and the life expectancy for that specific age.

Data analysis was done using Microsoft Office Excel version 2010 included all deaths in Malaysia in 2018. YLL was computed without age-weighting or discounting. Outliers and illogical data were identified and corrected during the data cleaning process. Ill-defined causes of death were reclassified and redistributed. The cause specific mortality fractions derived from verbal autopsy data were applied to non-medically certified deaths data for quality improvement.

To enable comparisons of YLL across different age and sex groups, the rate of YLL per 1000 individuals in the population was calculated. The YLL rate was calculated by dividing the total YLL by the population size in each age and sex group and then multiply by 1000.

## Ethics approval

The study was conducted in accordance with the Declaration of Helsinki and the Malaysian Guideline for Good Clinical Practice. It was approved by the Medical Research and Ethics Committee, Ministry of Health Malaysia (NMRR-21–1355–60662).

## Results

### Pattern of YLL by age groups and sex

**Fig. 1:**
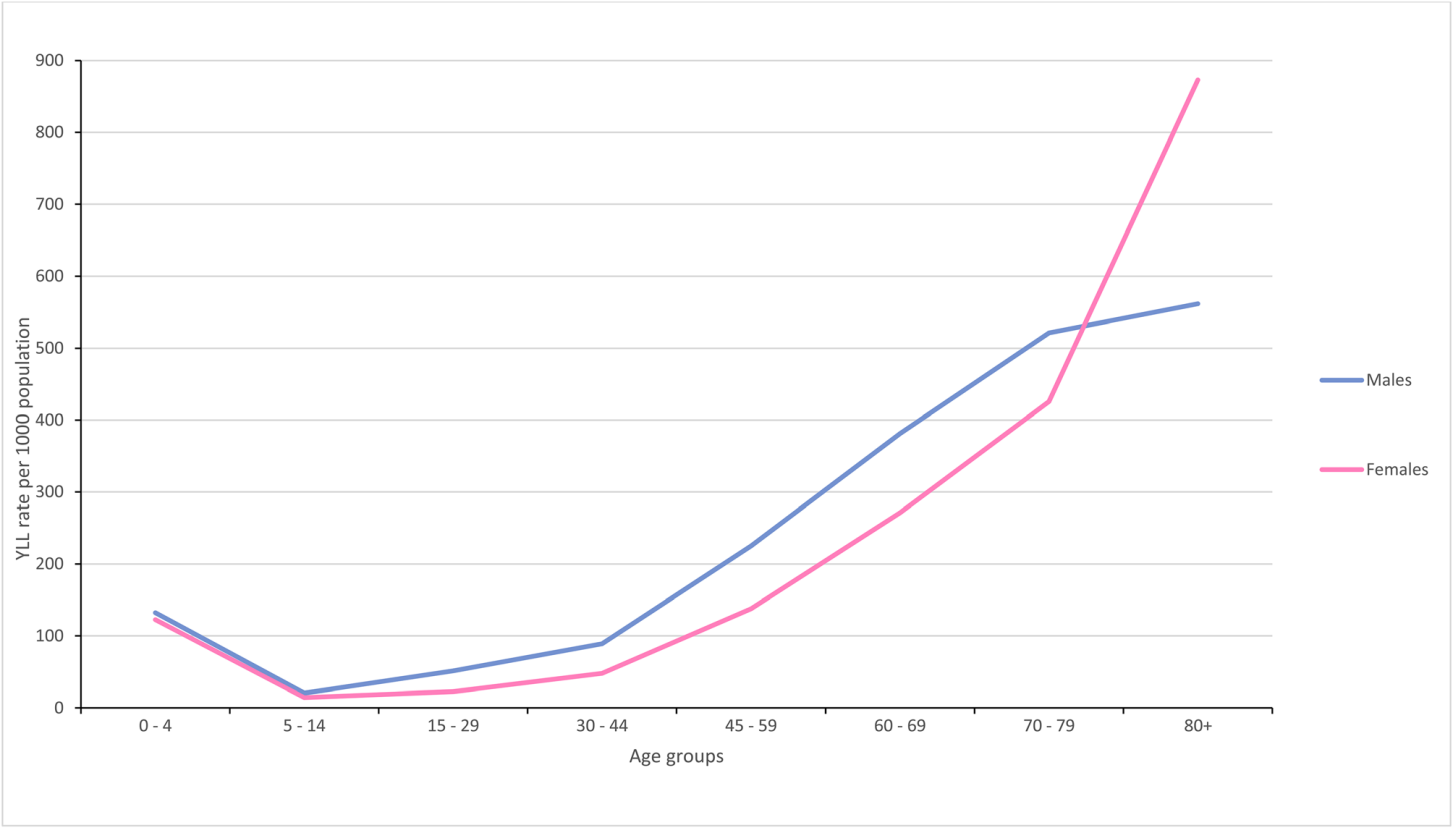
Year of life lost (YLL) rates by sex and age group, Malaysia, 2018

In Malaysia, premature mortality resulted in a total of 3.5 million YLL in 2018. Males contributed towards 2.1 million YLL, whereas females 1.4 million YLL. It was shown that the number of YLL increased with age for females, except age less than 5 years. The most significant increase in YLL was reported in females between the age of 70–79 and ≥ 80. In contrast, YLL for males increased slightly in the 70–79 and ≥ 80 age groups. Except for ≥ 80 age group, males had more YLL rates than females (Fig. 1).

## Pattern of YLL by broad cause group

Table 1 shows the YLL among Malaysian adults in 2018 by sex and broad cause group. The YLL rate per 1000 population was 107.2, with males having a rate of 123.2 YLL per 1000 population and females having 90.1 YLL per 1000 population. In all three broad groups, males had greater YLL rates per 1000 population than females. The largest YLL was in Group II (72.2%), followed by Group I (16.9%) and Group III (10.9%).

**Fig. 2:**
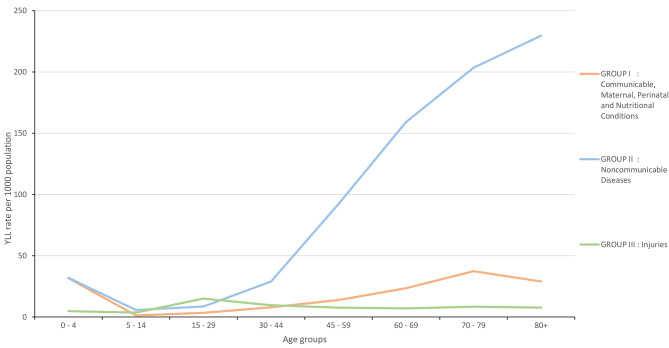
Year of life lost (YLL) rates by three broad group and age group, Malaysia, 2018

Figure 2 illustrates the YLL rates for three broad groups across various age groups. The number of YLL was seen to increase with age for Group I and Group II between the ages of 5–14 and 70–79, with the highest mortality burden occurring in the age of 80 years and above in Group II. However, there was a drop in YLL between the age groups of 70–79 and ≥ 80 in Group I. For group III, the number of YLL increased with age groups between 5–14 and 15–29, but reduced after age 30 years.

**Table 1:** Years of life lost (YLL) by sex and broad cause group in Malaysia, 2018

Broad cause group	Total			Males			Females				
	YLL	%	Rate/1000	YLL	%	Rate/1000	YLL		%	Rate/1000	
Group I	586,763	16.9	18.1	334,034	16.2	20.0	252,729		17.9	16.1	
Group II	2,506,044	72.2	77.4	1,423,257	69.1	85.1	1,082,788		76.7	69.1	
Group III	377,857	10.9	11.7	302,437	14.7	18.1	75,419		5.3	4.8	
Total	3,470,664	100.0	107.2	2,059,728	100.0	123.2	1,410,936		100.0	90.1	

## YLL by cause categories

**Fig. 3:**
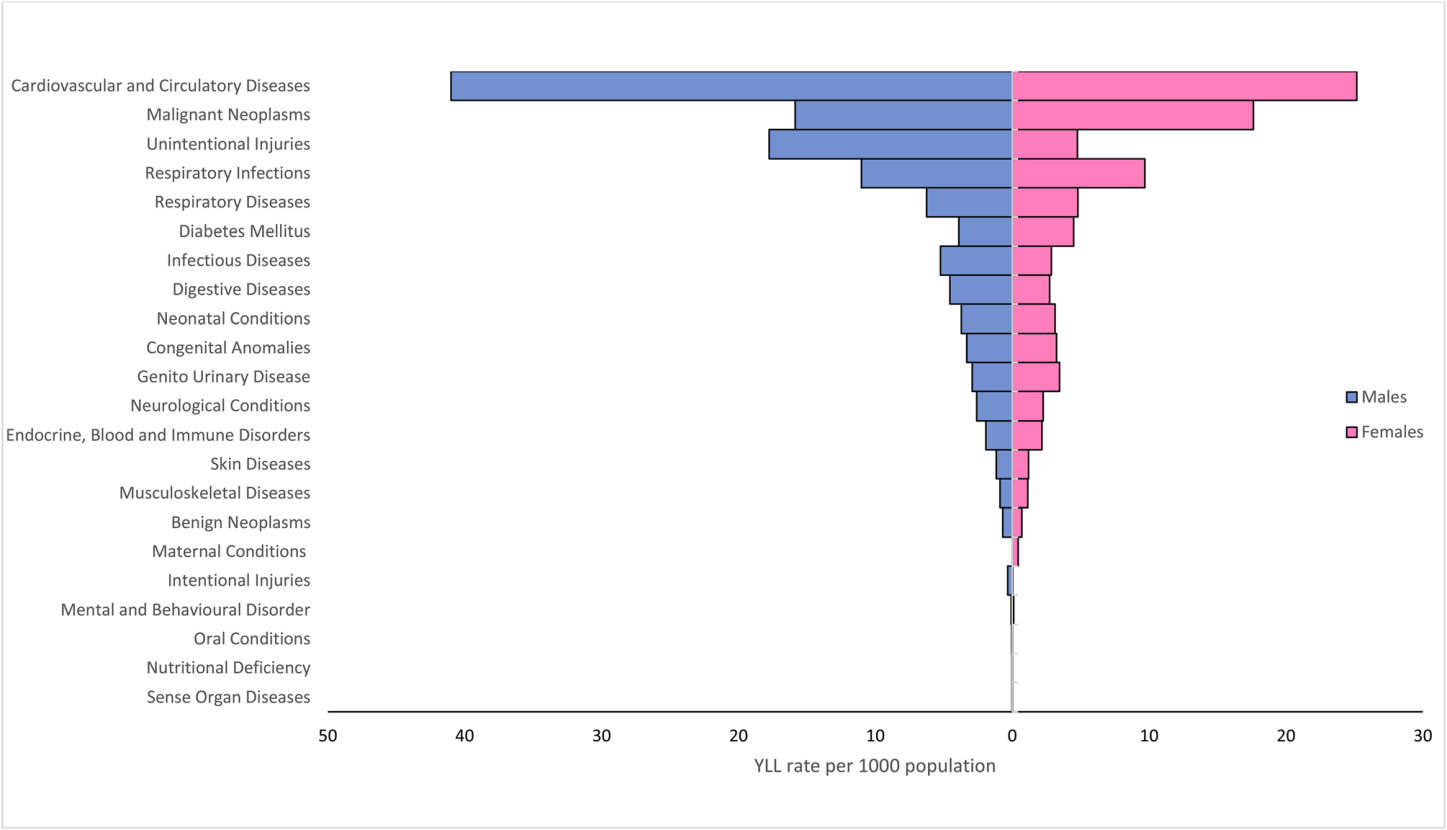
Years of life lost (YLL) rates by cause category and sex group, Malaysia, 2018

The burden imposed by cause categories (YLL per 1000 population) are shown in Fig. 3. The leading cause category of YLL in both males and females was cardiovascular and circulatory disease (41.0 YLL per 1000 population in males and 25.2 YLL per 1000 population in females), followed by unintentional injury (17.8 YLL per 1000 population) and malignant neoplasm (15.9 YLL per 1000 population) in males, and by malignant neoplasm (17.6 YLL per 1000 population) and respiratory infections (9.7 YLL per 1000 population) in females. YLL for cardiovascular and circulatory diseases, unintentional injuries, respiratory infections and respiratory diseases were all higher in males, whereas malignant neoplasms accounted for slightly more YLL in females.

### YLL by specific diseases

The top twenty specific diseases that caused the most YLL in Malaysia in 2018 are listed in Table 2. Ischaemic heart disease (17.7%) was the specific disease that led to the most YLL, followed by lower respiratory infections (9.7%), road traffic injuries (8.7%), cerebrovascular disease (stroke) (8.0%), diabetes mellitus (3.9%), trachea, bronchus and lung cancers (2.4%), breast cancer (2.2%), colon and rectum cancers (2.1%), nephritis and nephrosis (2.1%) and endocrine, blood and immune disorders (1.9%). Calculating YLL by sex revealed differences in the burden of premature mortality between men and women. Table 2 shows the most common specific diseases that led to the highest YLL by sex. For both sexes, the leading specific diseases in terms of premature deaths were ranked differently. Ischaemic heart disease (20.6%) ranked first in males, followed by road traffic injuries (11.2%), lower respiratory infections (8.9%), cerebrovascular diseases (8.0%), and diabetes mellitus (3.2%). In females, ischaemic heart disease (13.4%) and lower respiratory infections (10.7%) were the major causes of YLL, followed by cerebrovascular diseases (9.7%), breast cancer (5.4%), and diabetes mellitus (5.0%).

**Table 2:** Top 20 leading cause of Years of life lost (YLL) by specific diseases, Malaysia, 2018

	Total				Males				Females			
Specific diseases	Rank	YLL	%	Rate/ 1000	Rank	YLL	%	Rate/ 1000	Rank	YLL	%	Rate/ 1000
Ischaemic Heart Disease	1	612,751	17.7	18.9	1	423,792	20.6	25.3	1	188,958	13.4	12.1
Lower Respiratory Infections	2	335,077	9.7	10.3	3	183,564	8.9	11.0	2	151,513	10.7	9.7
Road Traffic Injuries	3	301,808	8.7	9.3	2	230,943	11.2	13.8	6	45,896	3.3	2.9
Cerebrovascular Diseases (Stroke)	4	276,840	8.0	8.5	4	164,903	8.0	9.9	3	136,905	9.7	8.7
Diabetes Mellitus	5	135,406	3.9	4.2	5	65,158	3.2	3.9	5	70,248	5.0	4.5
Trachea, Bronchus and Lung Cancers	6	83,002	2.4	2.6	6	54,827	2.7	3.3	10	28,175	2.0	1.8
Breast Cancer	7	76,165	2.2	2.4	-	344	0.0	0.0	4	75,821	5.4	4.8
Colon and Rectum Cancers	8	73,143	2.1	2.3	7	40,431	2.0	2.4	9	30,773	2.2	2.0
Nephritis and Nephrosis	9	71,204	2.1	2.2	8	36,715	1.8	2.2	7	36,428	2.6	2.3
Endocrine, Blood and Immune Disorders	10	66,148	1.9	2.0	10	32,123	1.6	1.9	8	34,025	2.4	2.2
Liver cancers	11	48,285	1.4	1.5	9	34,630	1.7	2.1	18	13,655	1.0	0.9
Congenital Heart Diseases	12	39,302	1.1	1.2	14	21,003	1.0	1.3	12	18,299	1.3	1.2
Skin and subcutaneous diseases	13	38,254	1.1	1.2	15	19,444	0.9	1.2	11	18,810	1.3	1.2
Drowning	14	35,894	1.0	1.1	11	29,696	1.4	1.8	30	6198	0.4	0.4
Tuberculosis	15	34,463	1.0	1.1	13	24,102	1.2	1.4	20	10,361	0.7	0.7
Chronic Obstructive Pulmonary Disease	16	34,206	1.0	1.1	12	25,304	1.2	1.5	22	8902	0.6	0.6
Leukaemia	17	33,718	1.0	1.0	18	18,336	0.9	1.10	16	15,383	1.1	1.0
Low Birth Weight	18	32,230	0.9	1.0	19	18,030	0.9	1.1	17	14,201	1.0	0.9
Asthma	19	31,110	0.9	1.0	20	15,627	0.8	0.9	15	15,483	1.1	1.0
Pericarditis, Endocarditis and Myocarditis	20	27,914	0.8	0.9	17	18,646	0.9	1.1	21	9268	0.7	0.6

**Fig. 4:**
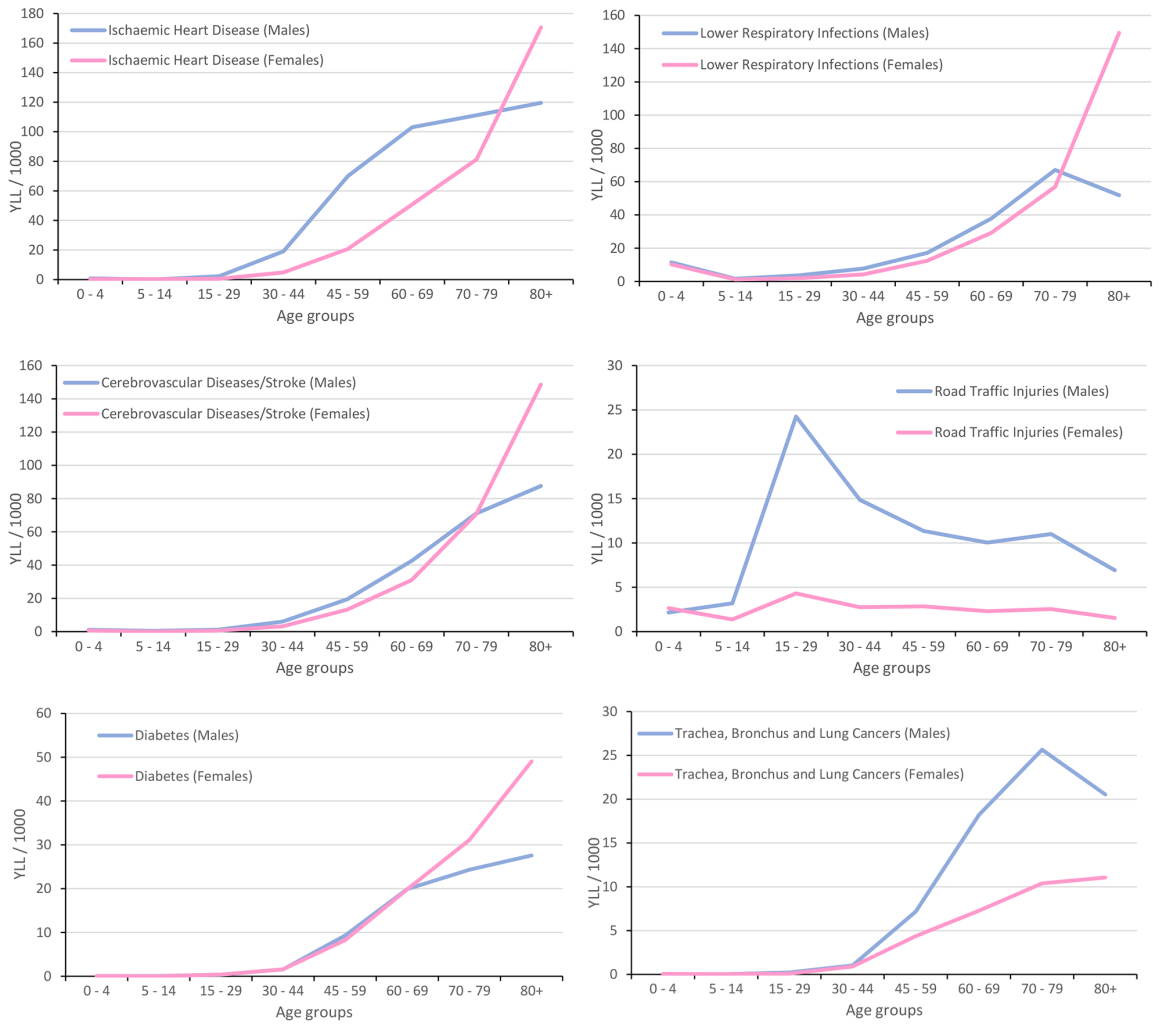
Distribution of years of life lost (YLL) rates by sex and age group among the leading specific diseases

YLL rates for six leading specific diseases are shown in Fig. 4 and are stratified by age and sex to indicate which age groups are most affected by the diseases. Starting at age 30, ischaemic heart disease exhibited an increase in YLL rates. Between the ages of 30 and 79, there was a significant sex difference, with males having greater YLL rates of ischaemic heart disease. Following that, a reversal trend was found, with females having higher YLL rates than males and peaking in the age-group over 80 years, with 170.6 YLL/1000 for females compared to 119.6 YLL/1000 for men.

Lower respiratory infections showed a similar pattern, with YLL rates steadily increasing beginning at age 30. Male YLL rates were slightly higher than females. A reversing trend was observed at age 80 and older, with 149.5 YLL/1000 for women compared to 51.8 YLL/1000 for men. Road traffic injuries, on the other hand, had a greater impact on the younger population aged 15–44 years, with a maximum of 24.2 YLL/1000 for men (age-group 15–29). As males aged, these rates decreased until age-group 60–69, but then rose at age-group 70–79.

Cerebrovascular disease and diabetes strongly affected the older persons population. At age 80 and above, cerebrovascular disease reached the maximum rates of 148.6 YLL/1000 for women and 87.5 YLL/1000 for men. Moreover, diabetes reaching the maximum rates of 49.0 YLL/1000 and 27.6 YLL/1000 for women and men in the age-group of over 80, respectively. The highest rates of trachea, bronchus and lung cancers were observed in people over 30 years, with maximum rates of 25.6 YLL/1000 for men aged 70–79 years and 11.1 YLL/1000 for women over 80 years. Rates were two times higher in the male population.

## Discussion

This study estimates the burden of premature mortality for diseases and injuries in Malaysia using the YLL measures defined in the GBD. Our findings demonstrate the public health significance of various diseases and injuries in premature deaths. In 2018, we estimated Malaysia’s premature death rate to be 107.2 YLL per 1000 population. The YLL measure was 1.51 million (0.92 million YLL in males and 0.59 million YLL in females) in 2008 [4]. In terms of causes of death, mortality increase from 124,857 to 172,031, between 2008 and 2018. Chronic diseases, such as heart disease and cancer, cause a rise in YLL. Moreover, an increased burden in NCDs warrants immediate policy attention.

Older adults exhibited a greater mortality burden compared to younger individuals. Males had a higher mortality burden, which was 46 per cent greater than females. Males recorded higher YLL rates for all leading causes than females did, except diabetes mellitus, breast cancer, and endocrine, blood and immune disorders. This pattern could be due to the male-female health-survival paradox; men have higher mortality rates than women across all ages [19]. Behavioural factors such as risk-taking (e.g., smoking and alcohol consumption) have also explained female-male differences in mortality [20]. In contrast, women aged 80 years and above showed higher mortality rates than men. The increased mortality burden resulting from ischaemic heart disease, lower respiratory disease, cardiovascular diseases, and diabetes mellitus, contributed to the excess female mortality burden. These specific diseases were the main contributors to the YLL in older adults in 2014 and 2019 and were found to be higher in females than males aged ≥ 80 years [5, 6].

Three broad cause groups of the YLL measure in Malaysia are similar to those reported in other Asian countries [7]. Group II, NCDs accounted for the biggest amount of YLL and caused 72.2% of total YLL in Malaysia. Group I, communicable diseases and maternal, perinatal and nutritional conditions resulted in fewer YLL. The lowest number of YLL due to Group III, injuries. In Malaysia, injuries only accounted for 10.9% of total YLL. Furthermore, the pattern of mortality burden shifted from a predominance of injuries at younger ages to a progressive increase in chronic diseases in older adults. NCDs were the major causes of death according to YLL measure, which is consistent with earlier research [4]. Similarly, the World Health Organization estimated that NCDs were responsible for 73% of Malaysian deaths [21]. Our results are consistent with other findings from previous studies, which found that NCDs remain the most prevalent group of diseases contributing to the burden of premature mortality [14–16]. Cardiovascular diseases and cancers were attributed to the greatest number of YLL. Regarding specific causes, ischaemic heart disease, lower respiratory infections and road traffic injuries had the highest burden of premature mortality. Promoting prevention, control, and rigorous evidence-based medical technologies and health policies in specific areas requires a concerted effort by health policymakers to prevent premature and preventable deaths from chronic NCDs.

Cardiovascular and circulatory disorders were the primary causes of premature mortality in both sexes due to ischaemic heart disease and cerebrovascular diseases (stroke). The National Health and Morbidity Surveys (NHMS) showed an increased trend in prevalence of NCDs risk factors such as diabetes mellitus, hypertension, and hypercholesterolaemia. The prevalence of diabetes increased from 11.2% to 2011 to 18.3% in 2019, hypercholesterolemia from 35.1% to 2011 to 38.1% in 2019, and hypertension from 32.7% to 2011 to 30.0% in 2019 [22]. This high prevalence of NCDs risk factors is expected to continue increasing NCDs’ burden and complications. Reducing exposure to NCDs risk factors is urgently necessary. The National Strategies Plan of Action for NCD (NSP-NCD) 2016–2025 is one of the initiatives dan action plans the government has implemented to reduce the burden of NCD in Malaysia [23]. In order to ensure the effectiveness of implementation, this strategy is implemented with other initiatives and action plans, such as the National Plan of Action for Nutrition of Malaysia (NPANM) III 2016–2025 [24] and the National Strategies Plan for Active Living 2016–2025 [25].

The second highest cause category contributing to YLL was malignant neoplasm. In both sexes, trachea, bronchus and lung cancer and colon and rectum cancers, were among the top 10 causes of death. Breast cancer was reported as the top four cause of death among females, while liver cancer ranked as the top nine cause of death among males. In Malaysia, various initiatives and programs have been implemented to reduce the burden of cancer, including the National Strategic Plan for Cancer Control Programme (NSP-CCP) 2016–2020 [26]. Furthermore, there is a need for public health intervention to increase cancer awareness among Malaysians aged 40 years and older who are at a higher risk for cancer [27]. To encourage regular health screening, health screening programmes, such as PeKa B40 health screening initiative for the low-income B40 group have been established and implemented to reduce the mortality and morbidity of cancers [28].

In 2018, the top disease-specific contributor of YLL among Malaysians was ischaemic heart disease. This is consistent with previous findings; between 2015 and 2017, ischaemic heart disease was the leading cause of death. The number of YLL due to this cause is growing, and in 2009, YLL was 323,821 (11.9%), compared to 612,751 (17.7%) in 2018. Due to worsening risk factors, it is expected that ischaemic heart disease mortality will rise in the next years. The increasing burden of cardiometabolic risk factors for heart disease, such as obesity, diabetes, hypertension, and high cholesterol, may contribute to the increased heart disease burden. Since the prevalence of abdominal obesity in Malaysia exceeds 52% [22], the best strategy to lessen the burden of heart disease could be to tackle these risk factors through public health policies. According to a local study published in 2020, there is a lack of awareness about risk factors for heart attack [29]. Only 5.6% of individuals identified all risk factors. In 2019, NHMS reported that only 33.3% of Malaysians had regular medical check-ups. These findings highlight the importance of the current healthcare protection scheme, as the Social Security Organization of Malaysia provides health screening for employees aged 40 years and above. In addition, the implementation of KOSPEN (Healthy Community Empowers the Nation) NCDs prevention programme [30] has to be improved and expanded.

Lower respiratory infections ranked 2nd in Malaysia in 2018 (ranked 2nd among females and 3rd among males). Lower respiratory infections, such as pneumonia and influenza, still appear to be significant diseases for the Malaysian population and should be addressed further through preventive measures. As a result, influenza vaccination should be promoted in the community, particularly among the older persons. On the other hand, there is a substantial decrease in road traffic mortality. Road traffic injury was the leading cause of fatal burden in 2009, with 12.8% of total YLL and it was dropped to the third leading cause of fatal burden in 2018 with 8.7% of total YLL. The reduction in road traffic fatalities in the population of Malaysians over these years is due to the Malaysian government having initiated many road safety programmes to improve road traffic, including Malaysia Road Safety Plan, Road Safety Regulation, and My Safe Road Program. Road injuries and fatalities, particularly among motorcyclists, continue to be a concern for the Malaysian population and should be tackled further by improving road infrastructure, educating motorcyclists and public, and implementing and enforcing traffic laws [31].

Besides, trachea, bronchus, and lung cancers were the primary causes of YLL, which is likely related to high prevalence of smoking in Malaysia. Although the prevalence of smoking had declined slightly from 23.1% to 2011 to 21.3% in 2019, the overall prevalence of smoking remained high, at 40.5% for men [32]. In 2019, NHMS study showed that the prevalence of second-hand smoke was 31.0% at home, 27.2% at work, and 48.1% at eateries [22]. The new smoking ban in all eateries and smoke-free rules in public places and workplaces can help reduce exposure to second-hand smoke in a range of worksite and community settings. Furthermore, studies have shown that workers subjected to a smoking ban tend to reduce their daily consumption of cigarettes. In addition, smoking cessation services, such as the ‘mQuit’ program should be strengthened in order to reduce the smoking prevalence [33]. These measures can help to lower the smoking-related disease burden and healthcare expenditures.

The study quantifies the leading causes of premature death among Malaysians in terms of YLL, providing crucial information for developing health policies and plans. However, the burden resulting from morbidity outcomes is not covered in this study. Therefore, future MBOD studies aim to calculate the YLD to estimate morbidity outcomes and provide a more comprehensive assessment of BOD by calculating DALY measure. Apart from that, there are some limitations to consider in this study. The accuracy of YLL estimations depends on the quality of the reported cause of death. Non-medically certified deaths are less reliable than medically certified deaths, which may lead to an undercount of the causes of death. Therefore, verbal autopsy must be expanded to cover the entire country to improve the estimation of causes of death.

### Implications of the study

The prominence of NCDs as the leading cause of YLL highlights the urgent need for targeted interventions to address these conditions. Ischaemic heart disease, which is responsible for a significant number of premature deaths, emphasizes the importance of preventive measures and initiatives to improve cardiovascular health among the population [34]. It is crucial that public health policies prioritize the prevention and control of NCDs, focusing on key strategies to promote healthier lifestyles, implement effective screening programs, and ensure accessible and high-quality healthcare services. For example, countries such as Finland have successfully implemented community-wide interventions to reduce the burden of ischaemic heart disease, including tobacco control policies, promotion of healthier diets and increased physical activity [35].

To combat the risk factors associated with ischaemic heart disease, such as smoking, poor diets, sedentary behaviour, and harmful alcohol consumptions, comprehensive strategies are essential. These strategies should include early detection of risk factors, timely treatment, and the promotion of lifestyle changes. Countries such as Canada, Australia, North America, the United Kingdom, and Switzerland have implemented screening programs for cardiovascular risk factors, including blood pressure and cholesterol checks, in order to identify individuals at high risk and provide appropriate interventions [36]. Furthermore, Singapore has implemented a comprehensive infrastructure to support preventive measures for diabetes, including the “War on Diabetes” initiative. This involves promoting healthy lifestyles, regular health screenings, and implementing workplace interventions to prevent and manage diabetes [37]. Moreover, it is imperative to address other health challenges that contribute to premature mortality, such as lower respiratory infections, road traffic injuries, stroke, and diabetes. This requires strengthening healthcare systems, improving access to quality care, enacting road safety policies, and enhancing infrastructure to support preventive measures and timely interventions. Several countries around the world have implemented effective road safety policies aimed to address the issue of road traffic injuries and achieving significant reductions in road traffic incidents, such as Sweden’s Vision Zero approach, Australia’s National Road Safety Strategy, New Zealand’s Safer Journeys strategy, and The Sustainable Safety approach in the Netherlands [38].

In addition to healthcare efforts, collaboration among different stakeholders is crucial for effectively addressing NCDs. This collaboration can involve government bodies, healthcare providers, community organizations, and other relevant entities. For instance, the Global Hearts Initiative, a collaborative effort by the World Health Organization and partner organizations, promotes the implementation of comprehensive strategies for NCDs, involving governments, healthcare systems, and communities across 12 countries [39]. By working together, these stakeholders can pool resources, knowledge, and expertise to develop comprehensive and sustainable solutions. In the context of Klang Valley in Malaysia, the involvement of transportation authorities, local councils, and other relevant stakeholders is particularly important in tackling road traffic injuries and promoting safe and sustainable transport options [40].

The findings of this study have important implications in term of economics, society, and health. Understanding the social factors that influence the burden of disease can help policymakers and healthcare professionals make informed decisions and allocate resources efficiently. This can result in cost savings by focusing on preventive measures and early interventions, thus reducing the economic burden associated with treatment and management of diseases. By identifying social factors such as income inequality, social support systems, and access to healthcare, interventions can be developed to improve overall health outcomes and reduce the burden of disease among marginalized populations [41].

### Suggestions for future research

The MBOD study can be improved by calculating the burden of disease at the subnational level. The findings will provide a more accurate assessment of health equity. The information obtained can then be utilised to guide national and state health policies and efforts targeted at improving community health. The current study on the burden of disease in Malaysia only provides information on the country’s current state of health; it did not identify any significant risk factors. Future research should consider population group characteristics, such as sex, age and state, as well as particular risk factors (e.g., smoking, drinking, physical inactivity, obesity, low fruit/vegetable intake, high cholesterol, high blood pressure) for disease burden. This will help in developing and designing effective risk-factor interventions for each population group, in order to reduce the burden of chronic diseases and death rates.

To conduct a national burden of disease study, a wide range of data is required for both morbidity and mortality. Demographic and health data on the incidence, prevalence, and duration of various diseases, as well as data on disability and mortality due to these conditions is typically required. These data are collected from censuses, vital registrations, population-based surveys, hospital records and disease registries. Although there are improvements or changes in the data available for MBOD mortality assessment, morbidity estimates for some diseases suffer from inconsistent data sources over time. The Institute for Health Metrics and Evaluation (IHME) GBD estimates are used to measure various sequelae because of the lack of data sources in Malaysia, especially morbidity and disability data. In future MBOD studies, it will be necessary to use regional estimates from literature review and local registries in order to generate MBOD estimates that have an impact on health policy. Moreover, it is also important to include stakeholders in the MBOD study process to ensure that the estimations apply to Malaysian context and thus the findings are translated into actionable steps towards improving health outcomes.

Determining DALY metric is a complex process that involves various choices in data processing, methodology, and presentation and interpretation of results. The use of the DALY metric, which combines the incidence approach for mortality and the prevalence approach for morbidity, has received criticism and has led to difficulties in interpretation of results. Continuous effort is required to promote the concept of burden of disease, ensure transparency in data and methodology, and effectively communicate results, including through visualization. Despite uncertainties, the DALY results are considered estimates of the burden of disease, providing a picture of current status and future changes in the burden of disease. Furthermore, it is important to regularly update the study findings in order to keep pace with changes in disease patterns over time. These estimates are valuable for policymakers as they allow for comparison of different health conditions, examination of the YLD and YLL components, and understand how disease burden changes over time provides valuable insights for policymakers, which can help prioritise public health policies and allocate resources effectively to address the most urgent health needs.

## Conclusions

This study highlights the analysis of YLL, which provides a systematic examination of premature deaths at a population level and is an important component in identifying disease areas that deserve more attention from health policy. NCDs are a significant health concern in Malaysia and are the primary contributors to the overall burden of disease. These results are important in guiding the national health systems on how to design and implement effective interventions for NCDs, as well as how to prioritise and allocate healthcare resources.

## Data Availability

For data protection purposes, the data used in this study cannot be accessed publicly. However, it can be obtained from the Department of Statistics, Malaysia upon reasonable request and with the necessary permissions.

## List of abbreviations

DALY: Disability-adjusted life year
DOSM: Department of Statistics Malaysia
YLL: Years of Life Lost
YLD: Years Lived with Disability
GBD: Global Burden of Disease
MBOD: Malaysian Burden of Disease
NCDs: Noncommunicable Diseases
NHMS: National Health and Morbidity Survey
